# Trends in Hypertension Diagnosis and Self-Reported Cases: A Retrospective Analysis of National Health Interview Survey (NHIS) Database

**DOI:** 10.7759/cureus.80655

**Published:** 2025-03-16

**Authors:** Awanwosa V Agho, Habibah L Jiwo, Tolulope B Oni, Olufunsho O Ayo, Prosper I Oghenebrume, Saluwa Nansimbi, Olga L Vera Colon, Sanaam Khan, Okelue E Okobi

**Affiliations:** 1 Internal Medicine, Mercy Catholic Medical Center, Darby, USA; 2 School of Public Health, Texas A&M University, Texas, USA; 3 Family Medicine, Obafemi Awolowo College of Health Science, Olabisi Onabanjo University, Sagamu, NGA; 4 Internal Medicine, Windsor University School of Medicine, Washington, USA; 5 Medical Education, Dnipropetrovsk Medical Academy, Dnipropetrovsk, UKR; 6 Cardiology, Alberta Health Services, Calgary, CAN; 7 Family Medicine, Larkin Community Hospital Palm Springs Campus, Hialeah, USA; 8 Family Medicine, IMG Research Academy and Consulting LLC, Homestead, USA; 9 Family Medicine, Larkin Community Hospital Palm Springs Campus, Miami, USA; 10 Family Medicine, Lakeside Medical Center, Belle Glade, USA

**Keywords:** adults, hypertension, nhis, prevalence, retrospective data analysis, trends

## Abstract

Background: Hypertension is a major public health issue, contributing significantly to morbidity and mortality. Understanding trends in hypertension diagnosis and self-reported cases can help inform strategies for prevention and management.

Objective: The objective of this study is to evaluate the trends in hypertension diagnosis and self-reported cases in the United States (U.S.) through the use of National Health Interview Survey (NHIS) data (2019-2023). In particular, the study analyzes the changes in the prevalence rates across the major demographics (race, age, and gender), socioeconomic (social vulnerabilities, education, and income) and geographical factors through the use of statistical modelling. This study seeks to recognize the key determinants that shape such trends and evaluate their implication with regard to targeted interventions and public health policies.

Method: Data from the NHIS (2019-2023) were examined, focusing on trends in hypertension prevalence based on demographic factors such as age, gender, race, nativity, and social determinants of health (e.g., social vulnerability, employment status, education level, and family income).

Result: Hypertension prevalence among U.S. adults remained consistently high. Age-adjusted rates were 27.0% in 2019 and increased slightly to 27.5% in 2023. Males showed higher hypertension rates (28.3% in 2023) compared to females (26.7%). Among age groups, the highest rates were observed in older adults: 54.3% for those aged 65-74 and 62.7% for individuals 75 years and older in 2023. Racial disparities persisted, with Black adults having the highest hypertension prevalence at 34.8% in 2023, while Asians had the lowest at 22.3%. Hypertension rates also varied with socioeconomic factors: individuals with lower income (28.4% for those below 100% Federal Poverty Level (FPL)) and lower educational attainment (40.5% for those without a high school diploma) had higher prevalence rates. Social vulnerability and employment status also influenced hypertension trends, with higher rates in individuals with high social vulnerability or non-employment.

Conclusion: Hypertension remains a persistent health issue, particularly among vulnerable populations. Targeted interventions are needed to address these disparities and reduce the burden of hypertension in the U.S.

## Introduction

Hypertension, or high blood pressure, is a global health concern affecting individuals across all age groups [1]. It serves as a major risk factor for several cardiovascular diseases, including heart attack, stroke, and kidney failure [2]. Although hypertension is preventable and manageable through lifestyle changes and medication, it remains a leading cause of morbidity and mortality worldwide [3]. The condition often develops silently, with many individuals remaining undiagnosed until complications arise [4]. Early detection and effective management are crucial in preventing the severe long-term effects of hypertension. With the continuing aging of the global population, enhancing hypertension awareness, diagnosis, and treatment is important for reducing the disease's burden on public health [1-5]. Presently, an estimated 1.28 billion adults worldwide have hypertension, with two-thirds in low- and middle-income countries and 46% unaware of their condition. Despite treatment efforts, only 42% are diagnosed and treated, and 21% have their hypertension under control [1,5]. The prevalence of hypertension increases with age, and its onset is influenced by factors such as genetics, lifestyle choices, and environmental conditions [2,4-6]. Moreover, it is noteworthy that various socioeconomic factors, including environmental stressors, education level/health literacy, and limited healthcare access, significantly contribute to the increasing prevalence of hypertension [1-6]. Such disparities have disproportionately impacted marginalized populations, resulting in high rates of hypertension (uncontrolled) and related cardiovascular complications [1-6].

The pathophysiology involves multiple mechanisms that contribute to increased vascular resistance and blood volume. One key factor is the dysfunction of the endothelium, which leads to reduced nitric oxide production and impaired vasodilation. This increases vascular tone, causing the blood vessels to constrict [6,7]. Additionally, the renin-angiotensin-aldosterone system (RAAS) plays a central role in regulating blood pressure by promoting vasoconstriction and sodium retention, which increases fluid volume and raises blood pressure [6-8]. Another contributor is sympathetic nervous system overactivity, leading to increased heart rate and vasoconstriction. Furthermore, genetic factors, obesity, and insulin resistance can exacerbate these mechanisms, leading to chronic high blood pressure. Over time, hypertension can result in damage to vital organs such as the heart, kidneys, and brain, increasing the risk of cardiovascular disease and stroke [7-9].

The National Health Interview Survey (NHIS) serves as a vital tool for assessing health trends across the United States (U.S.). Conducted annually by the Centers for Disease Control and Prevention (CDC), the NHIS collects data on a wide range of health indicators, including chronic diseases like hypertension. By analyzing NHIS data from 2019 to 2023, researchers can explore changes in hypertension diagnosis rates, self-reported cases, and associated demographic factors [10]. Therefore, the objective of this study is to evaluate the trends in hypertension diagnosis and self-reported cases in the U.S. through the use of NHIS data (2019-2023). In particular, the study analyzes the changes in the prevalence rates across the major demographics (race, age, and gender), socioeconomic (social vulnerabilities, education, and income) and geographical factors through the use of statistical modelling. This study seeks to recognize the key determinants that shape such trends and evaluate their implication with regard to targeted interventions and public health policies. The findings aim to provide actionable insights to policymakers and healthcare providers for designing targeted interventions and reducing the burden of hypertension, particularly in vulnerable populations.

## Materials and methods

Data sources and study design

This study utilized data from the NHIS conducted between 2019 and 2023. NHIS is a nationally representative, cross-sectional survey designed to collect health-related information from the civilian, noninstitutionalized population of the U.S. The study utilized a probability-based and multistage stratified sampling design, which ascertains representation across the various demographic groups, including geographic regions, age, race/ethnicity, and sex. This ascertains that the study findings are generalizable to the U.S. population. Also, this analysis focused on trends in hypertension diagnosis and self-reported cases, leveraging the large, standardized dataset available through NHIS.

Moreover, for the data processing and cleaning, the NHIS imputed dataset was used for the missing values, given that NHIS utilizes several imputation methods for key variables. However, for variables whose imputed values were unavailable, listwise deletion was performed in instances where missingness surpassed 5%, even as mean imputation for continuous variables was applied if the missingness was below 5%. Sensitivity analysis was also performed to compare the results of the complete-case analysis and the imputed data set to ascertain robustness.

Study participants and questionnaires

The study included adult participants aged 18 years and older who responded to questions about hypertension diagnosis. Respondents who self-reported a prior diagnosis of hypertension, as determined by a healthcare provider, were included in the analysis. The analysis is based on household interviews conducted within a sample of the civilian, noninstitutionalized population. Estimates were derived from responses from the sample adults. The participants were excluded if their responses to the key survey questions were found to be incomplete, and in instances where their health-related data and demographic data were missing. Exclusion criteria were incomplete responses to relevant survey questions and missing demographic or health-related data. The survey questionnaires covered various aspects, including demographic characteristics, health behaviors, and chronic disease diagnoses, ensuring comprehensive data collection.

Data collection and quality assurance

Data collection was conducted through in-person interviews by trained NHIS personnel. Interviews were conducted using a standardized survey instrument to ensure consistency across all respondents. To maintain data quality, NHIS employs rigorous interviewer training, validation checks, and monitoring protocols. Additionally, data were reviewed for completeness and accuracy before being released for public use. Missing data were addressed using imputation methods provided by NHIS, to ascertain that all gaps within the datasets were appropriately filled in a manner that minimizes potential biases while maintaining data integrity.

Variables of interest

The primary variables of interest included the prevalence of hypertension diagnoses and self-reported cases from 2019 to 2023. Secondary variables included demographic factors such as age (categorized into four groups), sex (female/male), race/ethnicity (American Indian or Alaska native only, Asian only, Black only, White only, Native Hawaiian or other pacific islander only, American Indian or Alaska native and White, Black and White), Hispanic or Latino origin and race (Hispanic, Mexican/Mexican American, Non-Hispanic, Black only non-Hispanic, White only non-Hispanic, Other races non-Hispanic), nativity (US born/foreign born) and geographic variables included CDC social vulnerability status index (Little to no, low, medium, high social vulnerability) while socioeconomic factors such as educational level (less than high school diploma, High school diploma, Some college, college degree or higher), family income (Less than 100% FPL, 110% to less than 200% FPL, 200% and greater FPL), and employment status were also analyzed. Other covariates included health behaviors like physical activity, smoking status, and body mass index (BMI). These variables were used to explore potential disparities and trends over the study period.

Data analysis and statistical methods

Descriptive statistics were used to summarize the prevalence of hypertension diagnoses and self-reported cases across survey years. Trends over time were assessed using age-adjusted prevalence rates. Confidence intervals (CIs) were calculated to assess precision. Further, all analyses were performed using IBM SPSS Statistics 30.0 (IBM Corp., Armonk, NY), using key procedures that included CROSSTABS for the chi-square tests, WEIGHT BY for adjustment of the NHIS survey, and UNIANOVA for two-way ANOVA. Notably, the inferential data analysis was performed using the Chi-square test and two-way ANOVA to evaluate the statistical significance of associations between categorical and continuous variables. A significance level of 0.05 was used, with p-values less than 0.05 indicating statistical significance.

Ethical considerations

The NHIS data are de-identified and publicly available, ensuring confidentiality of participant information. Ethical approval for this analysis was not required as it involved secondary data analysis of a publicly accessible dataset. The study adhered to the ethical principles outlined in the Declaration of Helsinki for research involving human subjects.

## Results

The age-adjusted prevalence (Percentage ±95% CI) of hypertension among adults aged 18 years or older showed a gradual increase. In 2019, the prevalence was 27.0 ± 1.3%, decreasing slightly to 26.6 ± 1.3% in 2020. However, the prevalence rose steadily in the following years, reaching 26.9 ± 1.2% in 2021, 27.2 ± 1.3% in 2022, and 27.5 ± 1.2% in 2023. This trend indicates a consistent, albeit modest, increase in hypertension prevalence over the study period. The CIs remained relatively stable, reflecting consistent data across the years.

Based on gender

The trends in hypertension prevalence by gender showed slight variations for males and females. Among females, the prevalence was 26.9 ± 1.7% in 2019, decreasing to 26.2 ± 1.7% in 2020. It remained stable at 26.1 ± 1.7% in 2021, increased to 26.5 ± 1.6% in 2022, and slightly rose to 26.7 ± 1.6% in 2023. The differences in CIs from year to year were minimal, ranging from a CI difference of 0.7% (from 26.9% in 2019 to 26.2% in 2020) to 0.2% (from 26.5% in 2022 to 26.7% in 2023). For males, the prevalence was 27.2 ± 1.8% in 2019, slightly decreasing to 27.0 ± 1.8% in 2020 before rising to 27.7 ± 1.8% in 2021. The prevalence increased to 27.9 ± 1.8% in 2022 and 28.3 ± 1.7% in 2023. The differences in CIs across the years ranged from a 0.2% CI difference (from 27.2% in 2019 to 27.0% in 2020) to a 0.4% CI difference (from 27.9% in 2022 to 28.3% in 2023). Overall, the data show a modest increase in hypertension prevalence for both genders, with slightly higher values observed in males over the study period. The age-adjusted prevalence of diagnosed hypertension was significantly different for females over the years, with a p-value of <0.05. Table 1 presents the annual prevalence of diagnosed hypertension across various demographic characteristics such as gender, race/ethnicity, age groups, and nativity.

**Table 1:** Prevalence of diagnosed hypertension for adults by demographic characteristics U.S: United States, CI: Confidence interval

Characteristics	2019	2020	2021	2022	2023	P-value
Total 18 years or over, age-adjusted	27.0 ± 1.3	26.6 ± 1.3	26.9 ± 1.2	27.2 ± 1.3	27.5 ± 1.2	-
Gender: Age-adjusted prevalence of diagnosed hypertension for adults (Percentage±95% CI)						
Female	26.9 ± 1.7	26.2 ± 1.7	26.1 ± 1.7	26.5 ± 1.6	26.7 ± 1.6	<0.05
Male	27.2 ± 1.8	27.0 ± 1.8	27.7 ± 1.8	27.9 ± 1.8	28.3 ± 1.7	
Age: Age-adjusted prevalence of diagnosed hypertension for adults (Percentage±95% CI)						
18-44 years	7.4 ± 1.1	6.9 ± 1.2	6.7 ± 1.1	7.2 ± 1.1	7.4 ± 1.1	<0.05
45-64 years	34.3 ± 2.2	33.7 ± 2.3	34.1 ± 2.2	34.0 ± 2.2	35.1 ± 2.3	
65-74 years	54.9 ± 3.2	54.0 ± 3.2	54.3 ± 3.1	54.6 ± 3.2	54.3 ± 3.1	
75 years and over	64.5 ± 3.6	63.0 ± 3.8	63.4 ± 3.5	64.4 ± 3.6	62.7 ± 3.5	
Race: Age-adjusted prevalence of diagnosed hypertension for adults (Percentage±95% CI)						
American Indian or Alaska native only	31.3 ± 16.0	27.7 ± 12.0	29.2 ± 12.0	19.1 ± 9.8	25.1 ± 10.8	<0.05
Asian only	19.7 ± 4.7	20.6 ± 5.4	20.2 ± 4.4	21.1 ± 4.7	22.3 ± 4.8	
Black only	34.6 ± 3.8	34.6 ± 4.3	34.5 ± 4.0	34.4 ± 4.0	34.8 ± 4.1	
Native Hawaiian or Other Pacific Islander only	28.0 ± 22.2	22.6 ± 25.6	25.4 ± 18.8	14.5 ± 17.1	25.7 ± 25.6	
White only	27.1 ± 1.4	26.7 ± 1.4	27.3 ± 1.5	27.4 ± 1.5	27.7 ± 1.4	
American Indian or Alaska Native and White	29.9 ± 14.4	29.7 ± 16.2	32.3 ± 16.1	29.9 ± 17.4	34.3 ± 16.2	
Black and White	15.2 ± 15.6	20.1 ± 22.1	10.2 ± 10.8	17.8 ± 17.6	8.5 ± 11.0	
Hispanic or Latino origin and race: Age-adjusted prevalence of diagnosed hypertension for adults (Percentage±95% CI)						
Hispanic	20.2 ± 3.1	18.3 ± 3.1	18.8 ± 2.9	19.7 ± 2.8	19.9 ± 2.6	<0.05
Mexican/Mexican American	17.9 ± 4.0	17.4 ± 4.2	18.1 ± 3.9	18.6 ± 3.8	18.0 ± 3.4	
Non-Hispanic	28.4 ± 1.4	28.3 ± 1.4	28.5 ± 1.4	28.7 ± 1.3	29.1 ± 1.3	
Black only, non-Hispanic	34.7 ± 3.9	34.9 ± 4.2	35.2 ± 4.0	34.9 ± 4.1	35.2 ± 4.1	
White only, non-Hispanic	28.1 ± 1.5	27.9 ± 1.5	28.3 ± 1.6	28.6 ± 1.5	28.9 ± 1.5	
Other races, non-Hispanic	22.1 ± 4.0	22.2 ± 4.2	21.2 ± 3.7	21.1 ± 3.7	22.8 ± 4.0	
Nativity: Age-adjusted prevalence of diagnosed hypertension for adults (Percentage±95% CI)						
U.S.-born	27.9 ± 1.4	27.5 ± 1.3	28.0 ± 1.4	27.9 ± 1.4	28.3 ± 1.3	<0.05
Foreign-born	23.4 ± 3.1	22.6 ± 3.4	21.9 ± 2.9	24.1 ± 3.0	24.7 ± 3.1	

Based on age

**Figure 1:**
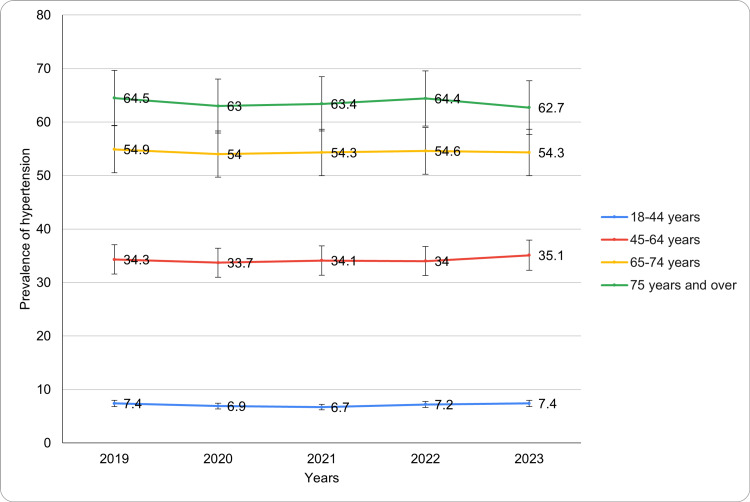
Diagnosed hypertension trends based on age during study period

The age-adjusted prevalence of hypertension among adults aged 18 years or older showed a gradual increase. In 2019, the prevalence was 27.0 ± 1.3%, and it decreased slightly to 26.6 ± 1.3% in 2020. However, the prevalence rose steadily in the following years, reaching 26.9 ± 1.2% in 2021, 27.2 ± 1.3% in 2022, and 27.5 ± 1.2% in 2023. This trend indicates a consistent, albeit modest, increase in hypertension prevalence over the study period. The CIs remained relatively stable, reflecting consistent data across the years. The analysis of hypertension prevalence by age group demonstrates notable differences across age categories (Figure 1). For adults aged 18-44 years, the prevalence of diagnosed hypertension remained relatively stable, starting at 7.4 ± 1.1% in 2019, decreasing slightly to 6.9 ± 1.2% in 2020, and reaching 6.7 ± 1.1% in 2021. It increased to 7.2 ± 1.1% in 2022 and returned to 7.4 ± 1.1% in 2023. Among individuals aged 45-64 years, the prevalence was 34.3 ± 2.2% in 2019, decreasing marginally to 33.7 ± 2.3% in 2020. It increased to 34.1 ± 2.2% in 2021, remained stable at 34.0 ± 2.2% in 2022, and rose to 35.1 ± 2.3% in 2023. For the 65-74 years age group, the prevalence was consistently high throughout the study period, beginning at 54.9 ± 3.2% in 2019 and slightly fluctuating with values of 54.0 ± 3.2% in 2020, 54.3 ± 3.1% in 2021, 54.6 ± 3.2% in 2022, and 54.3 ± 3.1% in 2023. In adults aged 75 years and over, the prevalence was the highest among all groups, at 64.5 ± 3.6% in 2019. It decreased to 63.0 ± 3.8% in 2020, increased slightly to 63.4 ± 3.5% in 2021 and 64.4 ± 3.6% in 2022, before dropping to 62.7 ± 3.5% in 2023. A significant change in the age-adjusted prevalence of diagnosed hypertension was observed over the years (p-value <0.05). These findings highlight the strong correlation between age and hypertension prevalence, with higher rates observed in older adults. The trends for diagnosed hypertension based on age have been presented in Figure 1. 

Based on race

**Figure 2:**
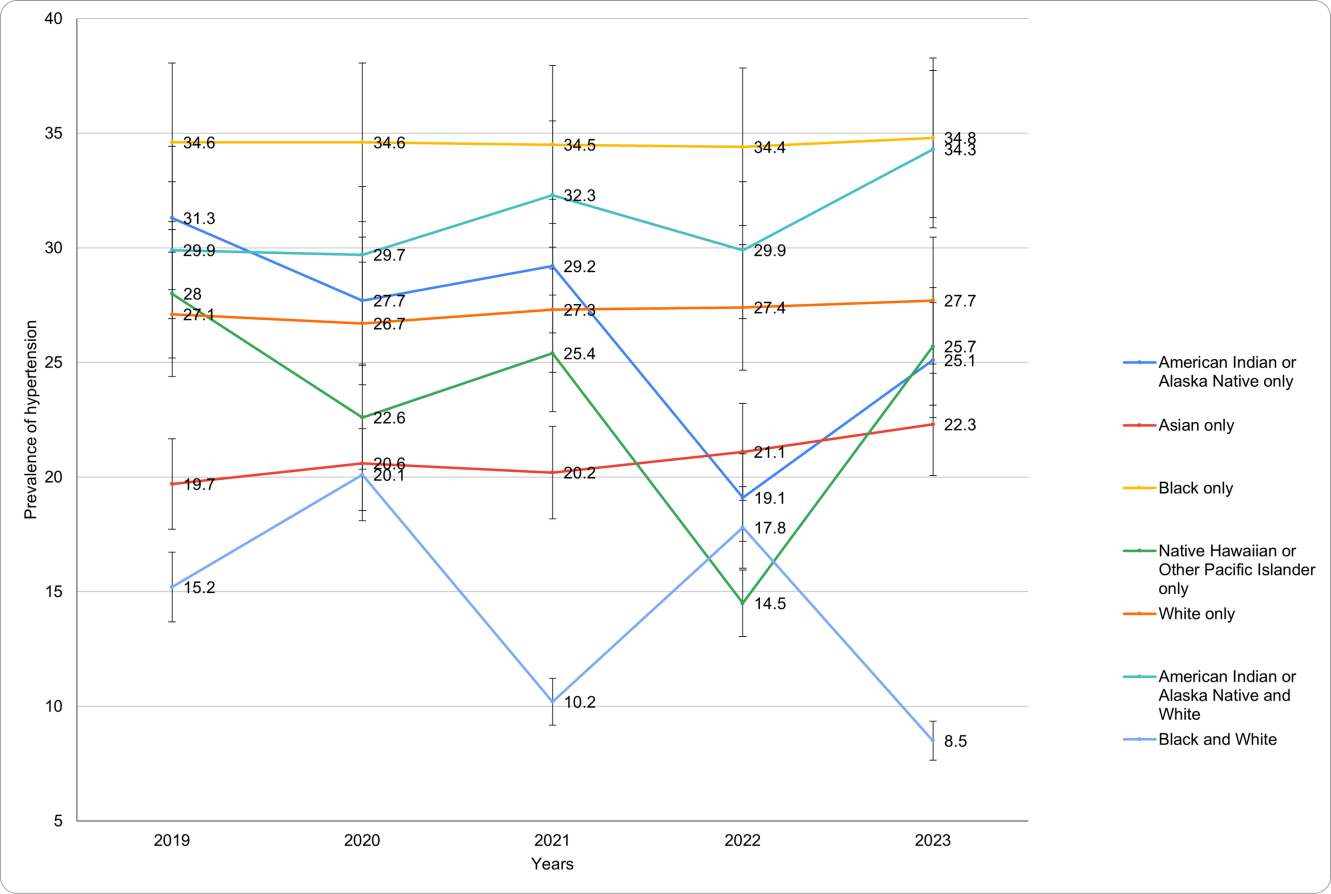
Diagnosed hypertension trends based on race during study period

The analysis of hypertension prevalence by race reveals considerable variation across racial groups (Figure 2). Among American Indian or Alaska Native individuals, prevalence started at 31.3 ± 16.0% in 2019, fluctuating over the years, with a notable decrease to 19.1 ± 9.8% in 2022 before rising to 25.1 ± 10.8% in 2023. Asian individuals exhibited a steady upward trend, increasing from 19.7 ± 4.7% in 2019 to 22.3 ± 4.8% in 2023. Black individuals consistently reported the highest prevalence, remaining stable at around 34.6 ± 3.8% in 2019 to 34.8 ± 4.1% in 2023. Native Hawaiian or Other Pacific Islanders showed significant variability, starting at 28.0 ± 22.2% in 2019, dropping to 14.5 ± 17.1% in 2022, and rebounding to 25.7 ± 25.6% in 2023. White individuals demonstrated relatively stable prevalence, increasing slightly from 27.1 ± 1.4% in 2019 to 27.7 ± 1.4% in 2023. Individuals of mixed American Indian or Alaska Native and White race exhibited an upward trend, rising from 29.9 ± 14.4% in 2019 to 34.3 ± 16.2% in 2023. In contrast, those identifying as Black and White displayed marked fluctuations, beginning at 15.2 ± 15.6% in 2019, peaking at 20.1 ± 22.1% in 2020, and declining to 8.5 ± 11.0% by 2023. There was a significant change in the prevalence over the years (p-value <0.05) across the racial category. The trends for diagnosed hypertension based on race have been presented in Figure 2. 

Based on Hispanic or Latino origin and race

The prevalence of hypertension among adults varied by Hispanic or Latino origin and race (Table 1). Hispanic adults exhibited a slight decline in hypertension prevalence from 20.2 ± 3.1% in 2019 to 19.9 ± 2.6% in 2023. Within this group, Mexican or Mexican American adults showed relatively stable rates, ranging from 17.9 ± 4.0% in 2019 to 18.0 ± 3.4% in 2023. In contrast, non-Hispanic adults reported higher and gradually increasing rates, from 28.4 ± 1.4% in 2019 to 29.1 ± 1.3% in 2023. Among non-Hispanic subgroups, Black adults consistently showed the highest prevalence, starting at 34.7 ± 3.9% in 2019 and rising slightly to 35.2 ± 4.1% in 2023. White non-Hispanic adults exhibited a modest increase from 28.1 ± 1.5% in 2019 to 28.9 ± 1.5% in 2023. Non-Hispanic adults of other races experienced a fluctuating trend, with prevalence ranging from 22.1 ± 4.0% in 2019 to 22.8 ± 4.0% in 2023.

Based on nativity

The prevalence of hypertension among adults showed distinct trends based on nativity (Table 1). U.S.-born adults consistently reported higher rates of diagnosed hypertension, starting at 27.9 ± 1.4% in 2019 and increasing slightly to 28.3 ± 1.3% in 2023. In contrast, foreign-born adults exhibited lower hypertension prevalence throughout the study period, with a slight fluctuation from 23.4 ± 3.1% in 2019 to 24.7 ± 3.1% in 2023. While the prevalence among U.S.-born individuals remained relatively stable, the rates among foreign-born individuals showed a modest increase after a dip in 2021. The prevalence of diagnosed hypertension among U.S.-born adults showed a significant change over the years (p-value <0.05). These findings highlight differences in hypertension trends by nativity status over the five-year period.

Based on the CDC social vulnerability index

**Figure 3:**
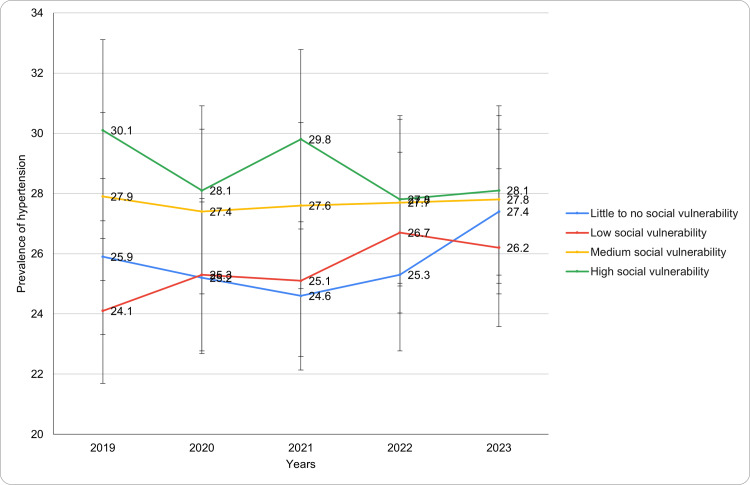
Diagnosed hypertension trends based on CDC social vulnerability index during study period

Table 2 presents the annual prevalence of diagnosed hypertension across various geographic characteristics like the CDC social vulnerability index, and socio-economic status such as employment status, education level, and family income for the years. The prevalence of hypertension varied across categories of the CDC Social Vulnerability Index (Figure 3). Among adults with little to no social vulnerability, rates ranged from 25.9 ± 2.8% in 2019 to 27.4 ± 3.2% in 2023, showing a slight increase. Those with low social vulnerability saw an upward trend, starting at 24.1 ± 2.5% in 2019 and peaking at 26.7 ± 2.6% in 2022 before slightly declining to 26.2 ± 2.7% in 2023. Adults with medium social vulnerability exhibited relatively stable rates, with minimal changes from 27.9 ± 2.2% in 2019 to 27.8 ± 2.2% in 2023. For individuals with high social vulnerability, hypertension prevalence initially decreased from 30.1 ± 2.7% in 2019 to 28.1 ± 3.1% in 2020 and stabilized at 28.1 ± 2.1% by 2023. A significant change in the age-adjusted prevalence of diagnosed hypertension was observed (p-value <0.05) across category. These findings reveal nuanced variations in hypertension prevalence across different levels of social vulnerability. The trends for diagnosed hypertension based on the CDC social vulnerability index during the study period have been presented in Figure 3.

Based on employment status 

The age-adjusted prevalence of diagnosed hypertension varied by employment status from 2019 to 2023 (Table 2). Employed adults showed a significant increase, from 18.3% ± 1.3 in 2019 to 19.1% ± 1.4 in 2023 (p-value <0.05). Adults not employed had a consistent prevalence of around 42.9% ± 2.3 in 2019 to 42.7% ± 2.3 in 2023. Full-time workers had a prevalence around 17.9% ± 1.4 in 2019, rising to 18.8% ± 1.5 in 2023. Part-time workers experienced a slight increase from 20.0% ± 3.3 to 20.2% ± 3.0. Those who had previously worked, but are not employed, had the highest prevalence, around 44%.

Based on educational level

**Figure 4:**
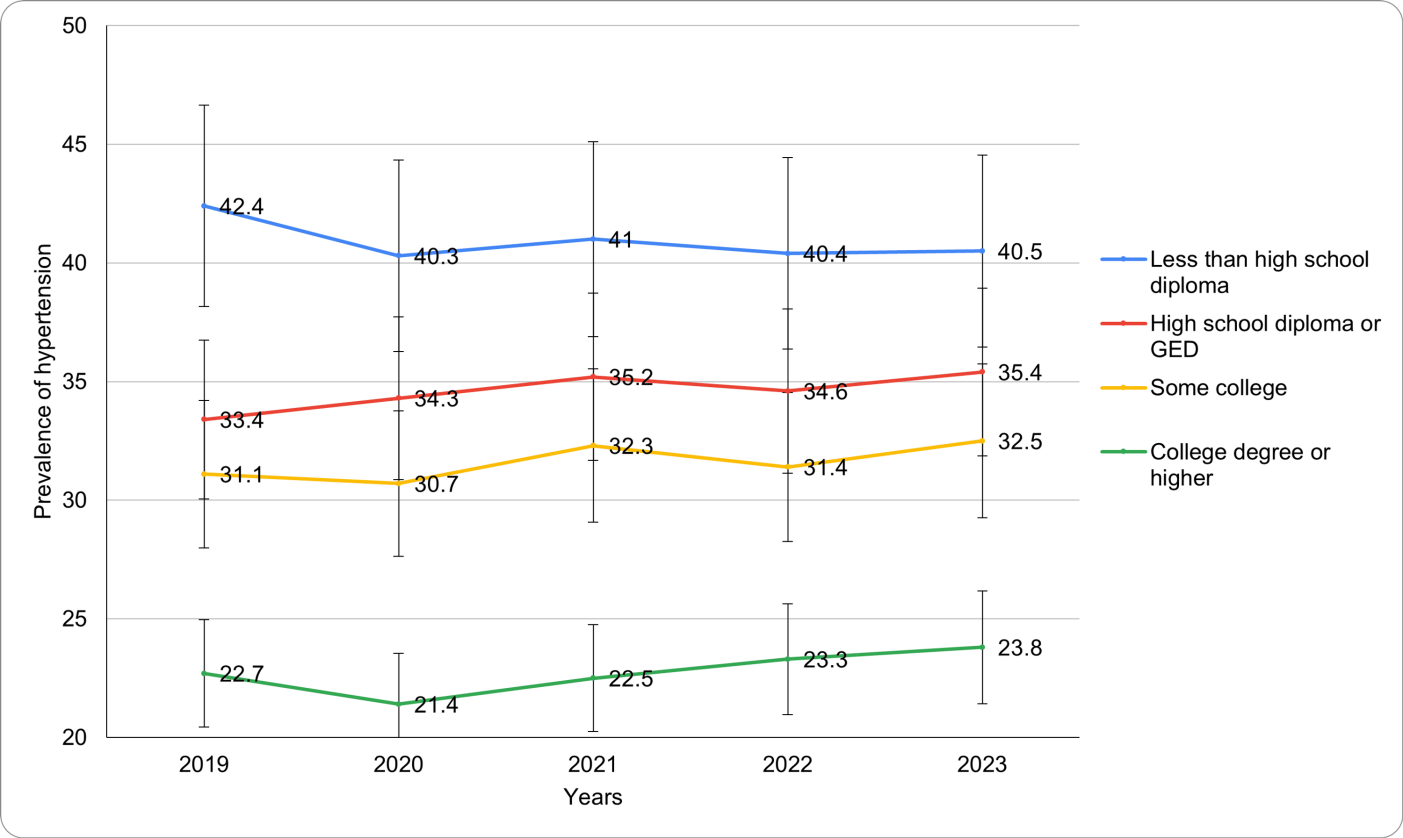
Diagnosed hypertension trends based on educational level during study period

Hypertension prevalence varied notably by educational attainment (Figure 4). Among individuals with less than a high school diploma, rates showed slight fluctuations, starting at 42.4 ± 4.5% in 2019 and decreasing to 40.5 ± 4.7% in 2023. Those with a high school diploma or GED demonstrated an increasing trend, from 33.4 ± 2.6% in 2019 to 35.4 ± 2.7% in 2023. Prevalence among individuals with some college education ranged from 31.1 ± 2.3% in 2019 to 32.5 ± 2.5% in 2023, showing moderate variation. For those with a college degree or higher, rates were lower overall, ranging from 22.7 ± 1.8% in 2019 to 23.8 ± 1.9% in 2023, reflecting a slight upward trend. A significant change in the age-adjusted prevalence of diagnosed hypertension was observed (p-value <0.05) across category. These findings underscore the inverse relationship between hypertension prevalence and educational attainment. The trends for diagnosed hypertension based on the level of education have been presented in Figure 4. 

Based on family income

Hypertension prevalence demonstrated variations across family income levels (Table 2). Among individuals with incomes less than 100% of the FPL, prevalence showed minor fluctuations, starting at 30.4 ± 4.0% in 2019 and declining to 28.4 ± 4.0% in 2023. For those with incomes between 100% and less than 200% FPL, rates remained relatively stable, ranging from 30.1 ± 3.2% in 2019 to 31.3 ± 3.0% in 2023, with a slight increase observed in the later years. Among individuals with incomes at or greater than 200% FPL, rates were consistently lower, starting at 25.7 ± 1.4% in 2019 and increasing slightly to 26.4 ± 1.4% in 2023. A significant change in the age-adjusted prevalence of diagnosed hypertension was observed (p-value <0.05) across category. These results indicate a trend where lower income is associated with higher hypertension prevalence.

**Table 2:** Prevalence of diagnosed hypertension by geographic characteristics and socioeconomic status for the time period CDC: Centers for Disease Control and Prevention, GED: General Educational Development program, FPL: Federal Poverty Level, CI: Confidence interval

Characteristics	2019	2020	2021	2022	2023	P-value
CDC Social Vulnerability Index: Age-adjusted prevalence of diagnosed hypertension for adults (Percentage±95% CI)						
Little to no social vulnerability	25.9 ± 2.8	25.2 ± 2.9	24.6 ± 2.7	25.3 ± 3.3	27.4 ± 3.2	<0.05
Low social vulnerability	24.1 ± 2.5	25.3 ± 2.7	25.1 ± 2.5	26.7 ± 2.6	26.2 ± 2.7	
Medium social vulnerability	27.9 ± 2.2	27.4 ± 2.3	27.6 ± 2.3	27.7 ± 2.5	27.8 ± 2.2	
High social vulnerability	30.1 ± 2.7	28.1 ± 3.1	29.8 ± 2.9	27.8 ± 2.2	28.1 ± 2.1	
Employment status: Age-adjusted prevalence of diagnosed hypertension for adults (Percentage±95% CI)						
Employed	18.3 ± 1.3	17.6 ± 1.4	18.0 ± 1.4	18.2 ± 1.4	19.1 ± 1.4	<0.05
Not employed	42.9 ± 2.3	40.8 ± 2.4	41.6 ± 2.1	42.9 ± 2.1	42.7 ± 2.3	
Full-time	17.9 ± 1.4	17.5 ± 1.5	17.7 ± 1.4	17.8 ± 1.5	18.8 ± 1.5	
Part-time	20.0 ± 3.3	18.3 ± 3.5	19.3 ± 3.2	19.5 ± 3.2	20.2 ± 3.0	
Not currently employed but has worked previously	44.9 ± 2.4	42.4 ± 2.3	43.4 ± 2.2	44.5 ± 2.1	44.3 ± 2.3	
Not currently employed and has never worked	23.1 ± 7.8	18.4 ± 7.9	18.7 ± 7.3	20.1 ± 9.0	21.2 ± 7.9	
Educational level: Age-adjusted prevalence of diagnosed hypertension for adults (Percentage±95% CI)						
Less than high school diploma	42.4 ± 4.5	40.3 ± 5.7	41.0 ± 4.8	40.4 ± 5.0	40.5 ± 4.7	<0.05
High school diploma or GED	33.4 ± 2.6	34.3 ± 2.8	35.2 ± 2.7	34.6 ± 2.8	35.4 ± 2.7	
Some college	31.1 ± 2.3	30.7 ± 2.4	32.3 ± 2.4	31.4 ± 2.6	32.5 ± 2.5	
College degree or higher	22.7 ± 1.8	21.4 ± 1.7	22.5 ± 1.8	23.3 ± 1.9	23.8 ± 1.9	
Family income: Age-adjusted prevalence of diagnosed hypertension for adults (Percentage±95% CI)						
Less than 100% FPL	30.4 ± 4.0	29.5 ± 4.5	30.5 ± 4.2	30.6 ± 4.4	28.4 ± 4.0	<0.05
100% to less than 200% FPL	30.1 ± 3.2	29.9 ± 3.5	29.2 ± 2.9	30.6 ± 2.9	31.3 ± 3.0	
200% and greater FPL	25.7 ± 1.4	25.4 ± 1.4	25.8 ± 1.4	25.9 ± 1.5	26.4 ± 1.4	

Overall, the results have indicated a modest but consistent increment in the prevalence rate of hypertension during the study period, alongside significant variations across the socioeconomic and demographic factors. Consistent hypertension prevalence has been noted to be higher in males than females, and this is attributable to biological differences, healthcare-seeking patterns, and various behavioral risk factors. Older persons, especially individuals aged 65 and above, indicated a significantly higher prevalence rate of hypertension, strengthening the established correlation between age and hypertension. The racial disparities have also been noted, with African Americans having a higher hypertension burden, indicating the possible influence of genetic predisposition, social health determinants, and access to healthcare. Moreover, individuals born in the U.S. presented a higher prevalence rate of hypertension than foreign-born persons, suggesting possible variations in dietary habits, healthcare access, and lifestyles. The different socioeconomic indicators, including unemployment, lower academic attainment, and low-income levels, have also been linked to a higher prevalence of hypertension, highlighting the role played by social vulnerability in hypertension burden. The observed trends offer an essential context for the interpretation of the findings of this study in the subsequent discussion section.

## Discussion

This study provides a detailed analysis of hypertension prevalence among U.S. adults, based on data from the NHIS. The findings highlight significant trends and variations in hypertension diagnosis and self-reported cases across various demographic characteristics, including gender, age, race, employment status, education level, family income, and social vulnerability. The following discussion will address the key findings from the data and compare these with existing literature on hypertension trends in the U.S.

Hypertension prevalence between males and females has remained relatively stable over the past five years. Male adults consistently reported a slightly higher prevalence of hypertension compared to females. In 2023, 28.3% of men reported being diagnosed with hypertension, compared to 26.7% of women. These findings are consistent with prior research conducted by Ostchega et al., which highlights a higher prevalence of hypertension among men during early adulthood (18-44 years), with the gender disparity diminishing in older age groups, particularly after 65 years of age [11]. This trend may be attributed to biological factors, as the study Xiang et al. conducted where they reported the cardioprotective effects of estrogen in premenopausal women, as well as the higher prevalence of cardiovascular risk factors observed in men [12].

Notably, even as sex differences have been acknowledged to play a major role, age remains a significant hypertension prevalence determinant with older persons experiencing considerably higher rates of hypertension. In 2023, the highest prevalence was recorded among individuals aged 75 and older (62.7%), a trend consistently observed throughout the study period. These findings align with previous research by Wang et al., which highlights the higher prevalence of hypertension in older populations due to age-related vascular changes. Laurent et al. further emphasize this association, reporting age-related vascular changes such as increased arterial stiffness [13,14]. Additionally, the prevalence among adults aged 45-64 years remains substantial (35.1% in 2023), underscoring the need for early detection and effective management of hypertension in middle-aged adults to mitigate long-term cardiovascular risks.

Interestingly, the prevalence of hypertension among younger adults (18-44 years) has remained relatively low, averaging around 7% throughout the study period. However, this group remains at risk due to increasing rates of obesity and sedentary lifestyles [14,15]. Ahmed et al.'s study highlighted a concerning trend, with hypertension-related mortality among young adults in the U.S. rising steadily until 2019, followed by a significant increase in 2020 and 2021. These findings emphasize the urgency of targeted strategies to address hypertension-related disparities and mortality in young adults. Early intervention is essential to mitigate risk factors and prevent the progression of hypertension as this population ages.

Significant racial and ethnic disparities in hypertension prevalence persist, with Black adults consistently demonstrating the highest rates of diagnosed hypertension over the years. In 2023, 34.8% of Black adults reported a hypertension diagnosis, compared to 27.7% among White adults [16]. These disparities are well-documented, as highlighted by Clark et al., who noted that Black populations are more likely to develop hypertension at a younger age and experience more severe forms of the condition compared to other racial groups [15,16]. Abrahamowicz et al. attributed these disparities to a complex interplay of genetic, environmental, and social factors, including limited access to healthcare, lower socioeconomic status, and lifestyle-related influences [17].

Hispanic adults, particularly Mexican/Mexican American individuals, exhibited a lower prevalence of hypertension compared to non-Hispanic Black and White, although the rates remained higher than those observed in younger populations. This finding is in line with research showing a complex relationship between hypertension and Hispanic ethnicity, influenced by factors such as acculturation, healthcare access, and differing health behaviors [15,18].

The analysis of hypertension prevalence by social vulnerability reveals critical insights. Adults residing in areas of high social vulnerability exhibited a higher prevalence of hypertension. In 2023, 28.1% of individuals in high social vulnerability regions were diagnosed with hypertension, compared to 25.2% in areas with minimal social vulnerability [19]. This finding aligns with the 2021 Reasons for Geographic and Racial Differences in Stroke (REGARDS) study, which underscored the strong association between social determinants of health-such as income, education, and healthcare access-and chronic disease prevalence, including hypertension. High social vulnerability is often associated with increased stress, unhealthy dietary patterns, reduced physical activity, and limited access to healthcare resources, all of which collectively elevate the risk of hypertension [19].

Employment status also plays a role in hypertension prevalence. Employed adults had a lower prevalence of hypertension (19.1% in 2023) compared to unemployed adults (42.7% in 2023). These findings may be attributed to differences in lifestyle, healthcare access, and socioeconomic status between employed and unemployed individuals [20]. Employed adults may have more stable incomes, better healthcare access, and a higher likelihood of engaging in health-promoting behaviors, such as physical activity and routine health check-ups [21]. Conversely, unemployment is associated with higher levels of stress and poorer mental health, which can contribute to hypertension development.

Educational level and family income are two key social determinants of health that influence hypertension prevalence. In 2023, individuals with less than a high school diploma exhibited the highest hypertension rates (40.5%), compared to those with a college degree or higher (23.8%). This trend is consistent with research indicating that lower education levels are associated with poorer health outcomes, likely due to limited health literacy, lower access to healthcare, and higher levels of stress [22]. Similarly, individuals with family incomes below the FPL experienced a higher prevalence of hypertension (28.4% in 2023), further supporting the role of socioeconomic factors in hypertension disparities [23,24].

The findings from this study emphasize the ongoing public health challenge posed by hypertension, particularly in vulnerable populations. While the overall prevalence has remained relatively stable over the five-year period, disparities based on age, gender, race, social vulnerability, education, and income continue to persist. These disparities highlight the need for targeted interventions to reduce the burden of hypertension, including public health campaigns aimed at increasing awareness, improving access to care, and promoting healthier lifestyles, particularly among high-risk populations such as Black adults, low-income individuals, and those with lower education levels [25,26].

Future research should focus on identifying the underlying causes of these disparities and exploring effective intervention strategies. For example, interventions to improve hypertension awareness, self-management, and access to care could help mitigate the effects of social vulnerability and lower socioeconomic status. Additionally, strategies to promote healthy behaviors such as physical activity, healthy eating, and stress management should be prioritized, especially for younger and middle-aged adults who are at risk of developing hypertension in later life [27,28].

Strengths and limitations

This study's strengths include its use of large, nationally representative NHIS data, providing robust estimates of hypertension trends across diverse demographic, socioeconomic, and health-related characteristics. The longitudinal analysis enables the identification of temporal patterns, highlighting disparities across race, age, gender, and social vulnerability indices. Additionally, age-adjusted prevalence rates ensure comparability across years, reducing confounding by demographic shifts. The detailed stratification by education, income, and employment offers valuable insights into the social determinants of hypertension.

However, the study has limitations. Self-reported hypertension may introduce recall bias and underreporting, particularly in populations with limited healthcare access or awareness. The absence of clinical confirmation (e.g., blood pressure measurements) limits the validity of the findings. The cross-sectional nature of NHIS data prevents causal inference. Additionally, variations in survey response rates and sampling weights over time may affect trend reliability. Finally, factors, like medication adherence and comorbidities, were not examined, which could influence hypertension rates.

Other potential limitations include the reliance on self-reported hypertension, which likely underestimates prevalence due to undiagnosed cases, and survey response bias, as NHIS non-response rates may skew estimates. Additionally, there is no discussion on confounders such as diabetes and obesity, which may influence hypertension trends. Another limitation is the lack of consideration for external factors that may have affected prevalence trends. The COVID-19 pandemic (2020-2021) likely reduced healthcare visits, leading to underdiagnosis, while health policy changes, such as Medicaid expansion and increased telemedicine use, may have also influenced trends.

## Conclusions

In conclusion, this study reveals significant trends and ongoing disparities in hypertension prevalence among U.S. adults. Hypertension remains a pressing public health issue, particularly affecting older adults aged 65 and above and disproportionately impacting racial and ethnic minorities, such as Black adults. Socioeconomic and social factors, including educational attainment, income, employment status, and social vulnerability, continue to shape these disparities, with disadvantaged populations facing a higher prevalence of the condition. These findings represent associations, not causation, and suggest longitudinal studies for causal inference. The findings emphasize the necessity of tailored interventions to address these inequities. Efforts should focus on enhancing public awareness, improving access to healthcare services, and promoting healthy lifestyle changes, particularly in high-risk groups. A multi-faceted approach is essential to reducing hypertension-related health burdens and advancing health equity across the U.S. population. Ongoing research and collaboration will be critical to achieving meaningful progress in combating this widespread condition.
